# Investigating the impact of diameters and thread designs on the Biomechanics of short implants placed in D4 bone: a 3D finite element analysis

**DOI:** 10.1186/s12903-023-03370-8

**Published:** 2023-09-22

**Authors:** Ali Robaian Alqahtani, Shrikar R. Desai, Jignesh R. Patel, Nasser Raqe Alqhtani, Abdullah Saad Alqahtani, Artak Heboyan, Gustavo V. O. Fernandes, Mohammed Mustafa, Mohamed Isaqali Karobari

**Affiliations:** 1 Department of Conservative Dental Sciences, College of Dentistry, Prince Sattam Bin Abdulaziz University, Al-Kharj, 11942 Saudi Arabia; 2 Department of Periodontology and Implantology, HKE’S S. Nijalingappa Institute of Dental Sciences and Research, Kalaburagi, 585105 India; 3 Dr. Patel’s Specialty Dental Care, 203, Silver Empire, Opposite Utran Power House Gate, VIP Circle, Utran, 394107 Surat India; 4 Department of Oral and Maxillofacial Surgery and Diagnostic Sciences, College of Dentistry, Prince Sattam Bin Abdulaziz University, Al-Kharj, 11942 Saudi Arabia; 5 Department of Preventive Dental Sciences, College of Dentistry, Prince Sattam Bin Abdulaziz University, Al-Kharj, 11942 Saudi Arabia; 6 Department of Prosthodontics, Faculty of Stomatology, Yerevan State Medical University, Mkhitar Heratsi, Str. Koryun 2, Yerevan, 0025 Armenia; 7 Department of Periodontics and Oral Medicine, University of Michigan School of Dentistry, 1011 North University Ave, Ann Arbor, MI 48109 USA; 8 Department of Restorative Dentistry & Endodontics, Faculty of Dentistry, University of Puthisastra, 12211 Phnom Penh, Cambodia; 9 Center for Global health Research, Saveetha Institute of Medical and Technical Sciences, Saveetha Medical College and Hospitals, Saveetha University, Kuthambakkam, India

**Keywords:** Bone quality, Immediate loading, Platform switched, Short implant

## Abstract

**Background:**

Dental implants emerge as a dependable and efficacious alternative for patients experiencing partial or complete tooth loss. The stability of these implants is influenced by surface topography and macro-level design. In cases where the height of the maxillary posterior region is diminished, employing short implants can prove advantageous. With the aim of examining the distribution of von Mises stress, strain, and micromovement in D4 bone quality surrounding platform-switched short implants, measuring 6 mm in length and featuring diameters ranging from 4 to 6 mm, as well as different thread designs, an in-depth finite element analysis was conducted under immediate loading conditions.

**Methodology:**

A 3D finite element model was constructed to simulate maxillary molar crowns, incorporating an implant with a length of 6 mm and varying diameters and thread designs. The diameters utilized were 4/3.6 mm, 5/4 mm, and 6/4.8 mm, while the thread designs included buttress, square, and triangle patterns. Each model underwent analysis with a 100 N force applied in two directions: vertical and oblique, relative to the long axis of the implant. Stress, strain, and micromovement in the peri-implant region were recorded, employing the Ansys Workbench R v.18.1 software for modelling and analysis.

**Results:**

When comparing all three diameters, the wide diameter (6 mm threads) exhibited the lowest values of peri-implant von Mises stresses (3.3 MPa and 35.1 MPa), strains (194 Ɛ and 484 Ɛ), and micromovements (0.7 μm and 1.3 Ɛ) subjected to axial and non-axial loading of a 100 N force. Notably, square microthreads yielded the most favorable stress parameters among the different thread shapes, manifesting the minimum values of stress, strains, and micromovements in their vicinity.

**Conclusion:**

For the treatment of atrophic ridges or in scenarios necessitating extensive surgical preparation of the implant site, a combination of short implants, wide diameters, and platform switching can be employed. In situations with reduced bone height and the requirement for an implant-supported prosthesis to replace a missing permanent maxillary molar, the utilization of wide-diameter platform-switched short implants measuring 6 mm in length, featuring a square thread design, should be taken into consideration.

## Introduction

Dental implants are an appropriate solution for partial or completely edentulous patients [1]. In order for an implant to function properly, it must be integrated with the bone. Multiple variables influence the interaction between bones and implants, such as interfacial bonding, the type, quantity, and quality of bone, bone metabolism, and the bone healing environment [2]. Additionally, occlusal loads are transmitted to peri-implant area [3] irrespective of the kind of prosthetics utilized for support. An implant’s ability to respond to force depends upon the BIC (how bone tissue will respond to mechanical forces) as well as the implant designs (shape, length, and diameter). Analyzing the functionality of prosthetic devices also requires consideration of the intensity, trajectory, and rate of the force application [4]. In addition, it is known that the implant’s micromotion must range between 10 and 150 μm, and excessive micromotion impairs the osseointegration and bone stability. This fact is crucial, independently of the implant design, and should always be observed, mainly because of early loading and immediate restoration of dental implants [5, 6].

Generally, the integration of implant grooves amplifies preliminary connection, enhance steadiness, and augment the extent of contact in equal measure. It is crucial to select the correct thread design for dental implants to achieve clinical success. In addition to offering optimal load distribution and protection against marginal bone loss, microthreaded implants preserve bone better than implants without microthreads [7–9]. Lee’s study found that microthreaded implants resulted in significantly less bone loss and more bone contact [10]. Approximately 50% of the implants within the microthread category made contact with the coronal portion of the bone, whereas the ratio surpassed 72.8% in the control group, signifying a notable difference [11]. In dental implant designs, several types of threads are used, including square threads, V-shaped threads, buttress threads, and reverse buttress threads [12].

The compactness of the bone greatly impacts the efficacy of dental implants. Typically, substandard bone, such as that located in the upper back jaw classified as D4-grade (a narrow band of solid bone encircling a mesh-like framework), exhibits a diminished rate of recuperation [13]. Inserting an implant within these areas can present difficulties. The study by Rungsiyakull P and colleagues focused on how bone types and loading patterns impact remodeling over 12 months in implant-supported single crowns. They considered stress, strain, strain energy density (SED), and bone density distribution. When applying an off-axial load to an implant, the highest von Mises stresses were observed in D4 (22.2 MPa) and D3 (21.9 MPa) bone categories [14]. There exist various alternatives to address the issue of subpar bone density, including augmenting the quantity of implants or employing a configuration with enhanced structural and site preparation features to enhance stability and contact area [14, 15]. Furthermore, it is possible to modify the implant design to minimize stress on softer forms of bone [16, 17]. In their research, Alemayehu D-B and colleagues employed Finite Element Analysis (FEA) simulations to delve into the effects of various thread designs and occlusal loading directions on the responses of both implants and the surrounding bone material. The study encompassed the evaluation of buccal-lingual, mesiodistal, and apical loading orientations. Study evaluated curved flank buttress and reverse buttress threaded implants and showed significant enhancements in the distribution of compressive stress, thereby substantially mitigating the levels of maximum stress experienced [18]. Udomsawat C and team analyzed stress around three dental implant designs during insertion into bone. They used dynamic finite element stress analysis. Results showed highest stress in cortical bone for all cases. Stress distribution varied based on implant geometry interacting with surrounding bone. [19].

Implant placement may have restrictions due to reduced bone height, independently if the density, due to anatomical structures [20, 21]. Therefore, extra-short, short, and wide implants have been recommended for those regions where there is not enough bone accessible for surgical insertion [22]. Short implants are typically placed in the alveolar bone with less height, offering several advantages over conventional implants in terms of cost, execution period, treatment requirements, and risks [23]. Moreover, the restorations with prosthetic components with a width smaller than the diameter of the implant may have less or no crestal bone resorption, favoring long-term success (platform switching) [24, 25]. Platform-switched implant systems create a right-angle step between an implant and an abutment. An inward-oriented implant–abutment connection facilitates the creation of a horizontal biological width, enhancing soft tissue attachment and reducing vertical bone resorption. Moreover, computational analysis results have indicated that platform switching can significantly reduce craniofacial stress by shifting the burden away from the connection between bone and implant [26]. Clinical research has shown that implant placements based on platform switching result in better soft and osseous tissue responses than implants paired to their platform [27, 28].

Analyzing biomechanics through the finite element analysis (FEA) method can be considered the most effective way to verify the stress distribution for implants [29]. Since dental implant–bone systems possess complex geometrical properties, FEA is widely recognized as the optimal approach for evaluating these constituents [30]. A significant obstacle for many researchers is the cost and complexity of the equipment needed for experimental testing. In comparison to experimental testing, the FEA method has the advantage of being more time efficient, simpler to use, and cheaper [31].

The design and form of dental implant threads can exert a noteworthy influence on oral biomechanics. To comprehend the impact of implant mobility on bone reaction, it is necessary to analyze the deformation of the BIC. Liu et al. [32], studying the macro thread designs of implants, reported that a short implant with a buttress thread generated reduced stresses in cancellous bone. Rismanchian et al. [33] noted in their study that straight/tapered triangular- or square-threaded implants had higher peak tensile and compressive stresses when compared to tapered implants with double-threaded triangular threads. On the contrary, Kong et al. [11] reported that square, buttress, reverse buttress, and V threads have better stress distribution depending on helix angle and width of the thread. It is, however, apparent in past literature that there is a need to ascertain the appropriate implant thread design suitable for proper distribution of stresses. The von Mises criterion is a suitable stress criterion for assessing the performance of ductile materials such as titanium. The von Mises formulation is deemed as a mathematically convenient and appealing approximation of the Tresca criterion [34]. While von Mises’s principle represents a limit of the stored deformative energy within the material, the Tresca principle indicates the material’s utmost shear stress [35]. Hence, the main objective of the present study was to evaluate the von Mises stress, strain, and micromovement dispersion by wide-diameter, platform-switched, short-length implants in D4 bone quality under immediate loading. Based on FEA simulations of distribution of stresses under load, the null hypothesis postulated in this study asserts that there are no notable distinctions between various diameters and thread configurations of short implants.

## Materials and methods

As a first step, virtual geometric models (VGMs) of D4 bones were generated with reference to Misch classification [36]. The results of VGMs are more reliable than those of actual structures. In order to assess structural and load characteristics from multiple angles, three-dimensional models were incorporated. In order to view and split CT images, Ansys R v.18.1 was utilized. A 3D tetrahedral structural solid finite element model of the implant, abutment, and surface of the biomaterial was constructed with Solid Edge, version 19. A computer with an Intel Core i9 processor and 32 GB of RAM running Windows 10 was used in this study. Each model took approximately 7 min to run. Solver sections are integral parts of FEA, which utilize algorithmic approaches to address a multitude of equations generated by preprocessors. A static Implicit solver was used in this study. Models were constructed using homogeneous, anisotropic, and proportionally resilient materials.

### Bone characteristics

**Fig. 1:**
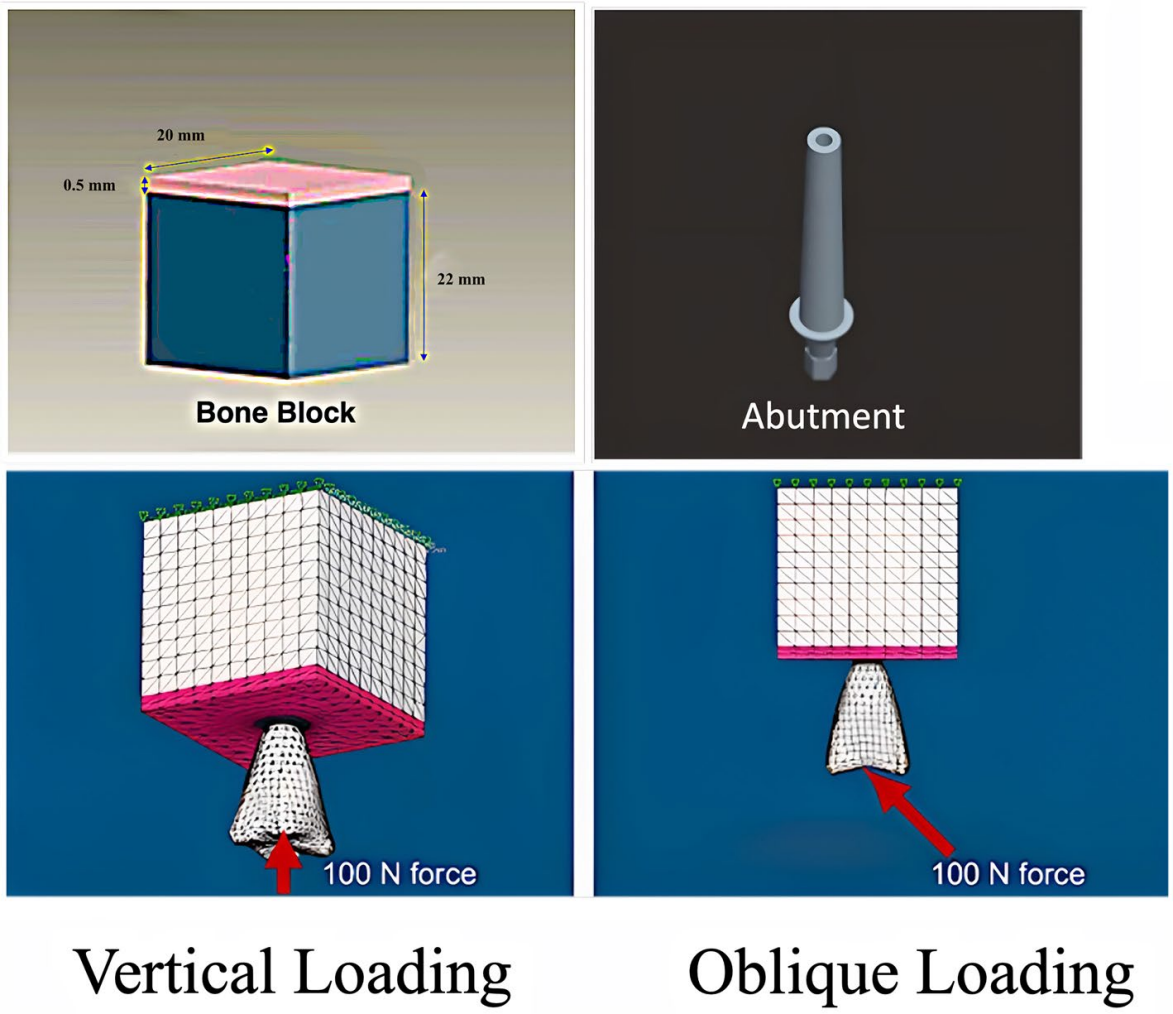
Bone block, Abutment and Loading application

The bone structure possessed a density value of 4 (referred to as D4) [36], showcasing a remarkable abundance of spongy bone. Its representation involved the following parameters: (i) vertical extent: 22 mm; (ii) horizontal span: 20 mm. A slender layer of 0.5 mm marked the magnitude of the outer compact layer of bone. Distinctive anisotropic properties were allocated to the cortical and cancellous constituents of this bone entity. (Fig. 1; Table 1) [15, 23].

**Table 1:** Material properties assigned to the model

S. No.	Material	Young’s Modulus (E MPa)	Poisson’s Ratio (v)	Shear Modulus (G MPa)
1.	Cortical Bone	Ex 12,600	V xy 0.300 V yz 0.253	G xy 4850
		Ey 12,600	V xz 0.253 V yx 0.300	G yz 5700
		Ez 19,400	V zy 0.390 V zx 0.390	G xz 5700
2.	Trabecular Bone	Ex 1148	V xy 0.055 V yz 0.010	G xy 68
		Ey 210	V xz 0.322 V yx 0.010	G yz 68
		Ez 1148	V zy 0.055 V zx 0.322	G xz 434
3.	Titanium	110,000	0.350	-
4.	Porcelain	70,000	0.190	-
5.	Cement	12,000	0.25	-
6.	Cobalt–Chromium Metal	87,900	0.30	-

### Implant setting

Each variation of the implant design consisted of a 6 mm-long anchoring component that integrated a support structure, along with a threaded section featuring either a buttress pattern or a triangular (V) configuration. The width of the threads measured 0.8 mm, while their height was 0.4 mm. All models were specifically developed to provide stability for a permanent maxillary first molar. The dimensions of the dental crown were determined based on the average measurements of the maxillary first molar, as indicated in Table 2. The screw exhibited a tapered shape by the 5th turn. The abutment’s height was set at 5 mm and it possessed an internal hexagonal connection, as illustrated in Fig. 1. The models featuring a diameter of 4 mm were designed with a platform-switching technique applied to 10% of the structure, whereas the models with 5 and 6 mm diameters incorporated a platform-switching technique applied to 20% of the structure.

**Table 2:** Dimensions assigned to the maxillary molar crown

1	Cervico-occlusal length	7.5 mm
2	Mesio-distal diameter	10.0 mm
3	Mesio-distal diameter at the cervix	8.0 mm
4	Buccolingual diameter	11.0 mm
5	Buccolingual diameter at the cervix	10.0 mm

### Loading

Each individual model underwent examination using a consistent force magnitude of 100 N, applied vertically (90 degrees) at the central fossa. Additionally, an inclined direction (45 degrees) relative to the long axis of the tooth (Fig. 1) was employed for immediate loading conditions at the bone-implant interface, considering a frictional coefficient of 0.3. The material characteristics employed in the models were obtained from existing research studies [24, 37] and appropriately allocated (Table 1).

### Meshed models

**Fig. 2:**
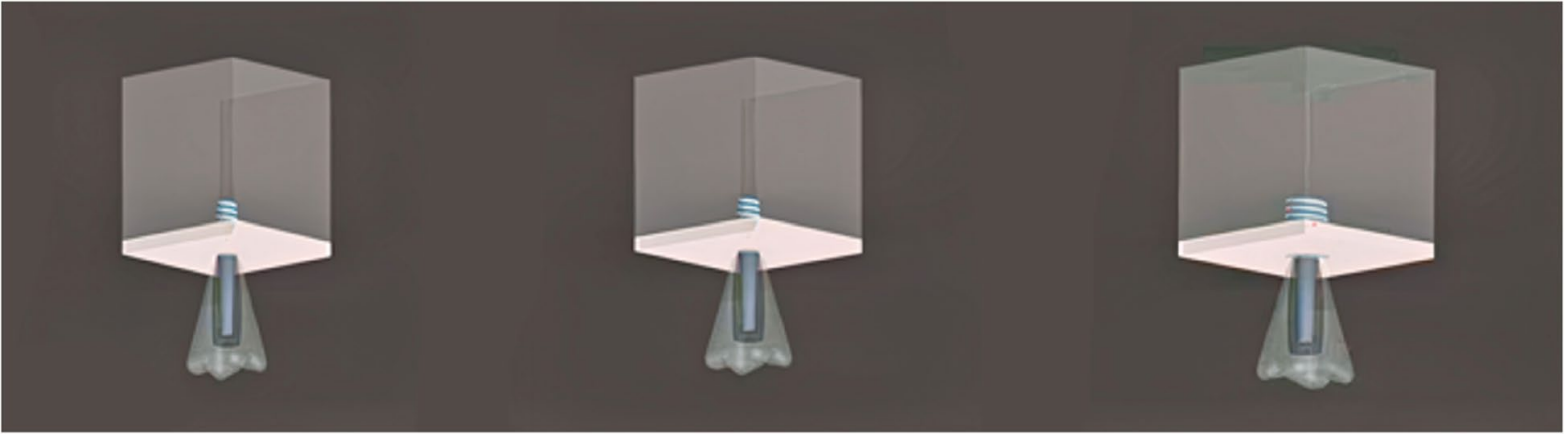
Buttress thread models of 4 mm (left), 5 mm (middle), and 6 mm diameter (right)

**Fig. 3:**
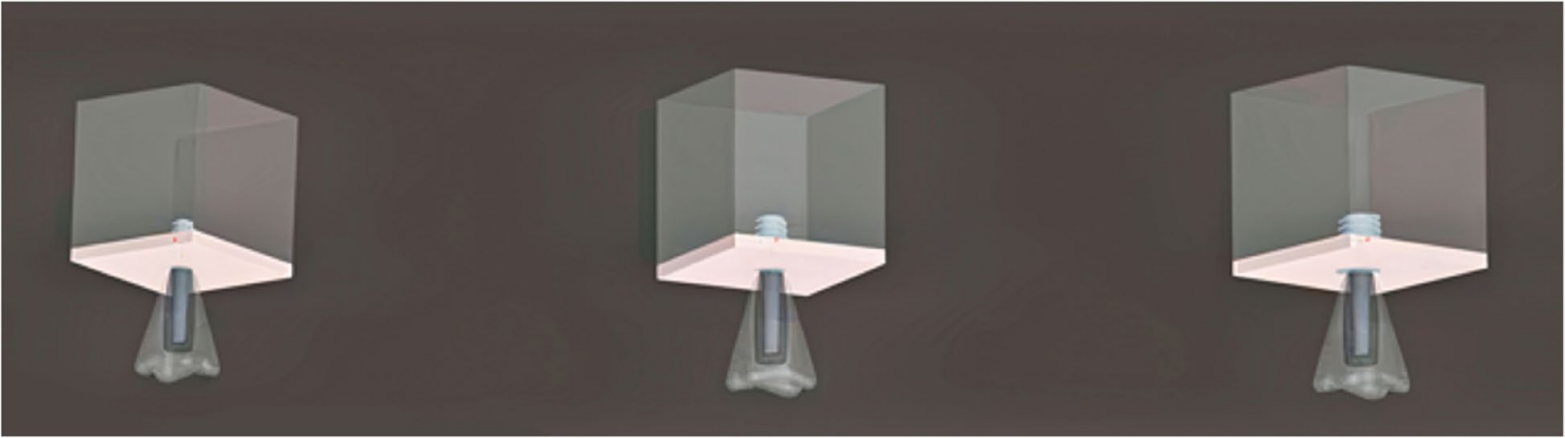
Square thread models of 4 mm (left), 5 mm (middle), and 6 mm diameter (right)

**Fig. 4:**
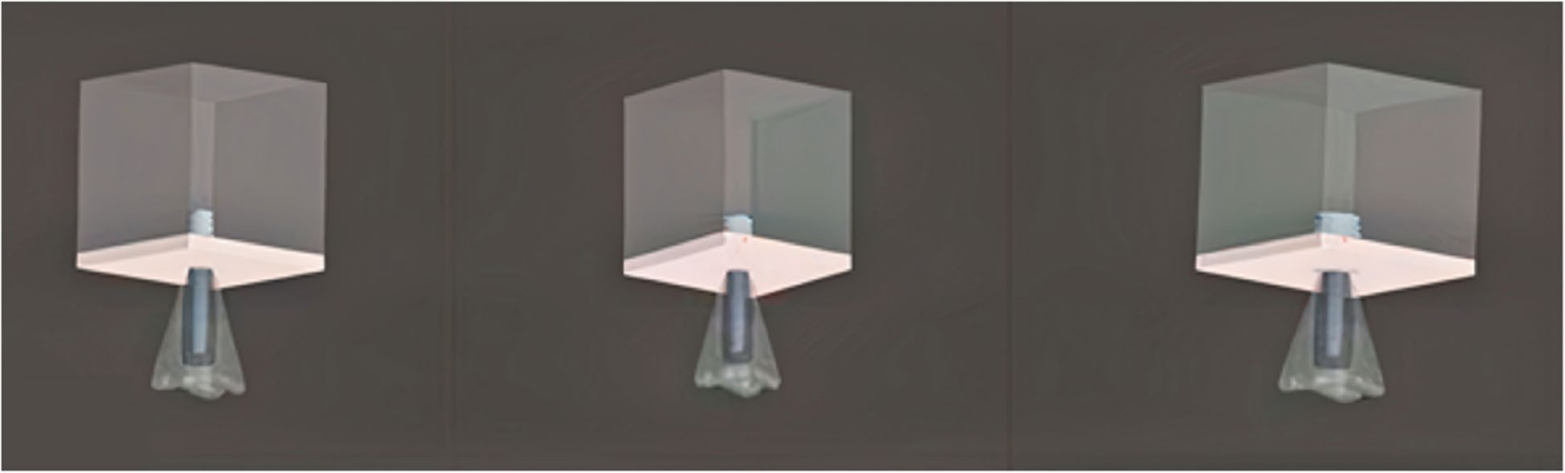
Triangular thread models of 4 mm (left), 5 mm (middle), and 6 mm diameter (right)

The 3D models underwent meshing using Hypermesh, v.13.0, employing a combination of tetrahedral and octahedral elements. The modeling process involved precise node positioning derived from mathematical calculations, considering the thread inclination (Fig. 1; Table 3). The numerical solutions of the elements directly correlate to the number of nodes and elements present. Tetrahedral elements, characterized by either four or ten nodes with three degrees of freedom per node (translations in the x, y, and z directions), were employed due to their versatility in handling intricate geometries. Specifically, ten-node tetrahedra were utilized to mesh the assembly. Given the structural complexity, hexamesh implementation was not viable, necessitating the adoption of a tetrahedral mesh with an element size of 0.5 mm. Boundary conditions were established by constraining all nodes at the base of the 3D models. The following models were designed: Model 1, buttress thread having a length of 6 mm and diameter of 4/3.6 mm; Model 2, buttress thread having a length of 6 mm and diameter of 5/4 mm; Model 3, buttress thread having a length of 6 mm and diameter of 6/4.8 mm (Fig. 2); Model 4, square thread having a length of 6 mm and diameter of 4/3.6 mm; Model 5, square thread having a length of 6 mm and diameter of 5/4 mm; Model 6, square thread having a length of 6 mm and diameter of 6/4.8 mm (Fig. 3); Model 7, triangle (V) thread having a length of 6 mm and diameter of 4/3.6 mm; Model 8, triangle (V) thread having a length of 6 mm and diameter of 5/4 mm; Model 9, triangle (V) thread having a length of 6 mm and diameter 6/4.8 mm (Fig. 4).

**Table 3:** Incorporated nodes and elements of the developed models

Diameter	Buttress		Square		Triangle	
	Nodes	Elements	Nodes	Elements	Nodes	Elements
4 mm	57,145	40,230	54,985	38,580	59,353	41,751
5 mm	63,095	44,637	61,611	43,409	63,559	44,880
6 mm	67,626	48,955	67,661	47,935	70,217	49,863

**Fig. 5:**
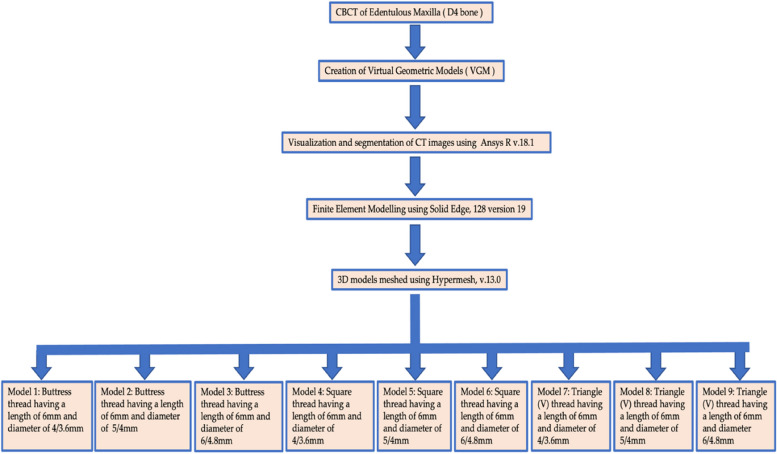
Flowchart depicting the study workflow

The interconnected models, known as finite element models, comprised vertices and elemental data, constructing a comprehensive framework for analysis. The accuracy of the finite element solution is directly correlated with the quantity of vertices and elements utilized, ensuring exact results. The assembly was methodically meshed using 10-node tetrahedral elements, providing a resilient representation of the structure. Given the intricacy of the system, a meticulously crafted tetrahedral mesh with an element size of 0.3 mm was implemented to capture intricate nuances. The mesh incorporated a substantial number of elements, totaling 784,386, and nodes, totaling 987,346, with a focus on refining regions prone to stress concentration. Key material properties, including Poisson’s ratio, Young’s modulus, shear modulus, and densities, were appropriately integrated into the mesh to accurately simulate the mechanical behavior. The modeling analyses were executed using Ansys R v.18.1, a powerful software package renowned for its capabilities in advanced numerical simulations and analysis (Fig. 5).

### Statistical analysis

After assessing the Gaussian distribution using the Shapiro-Wilk W test, it was confirmed by employing Levene’s test for homogeneity of variance on all collected data. Subsequently, in case of a normal distribution, the t-Student test was utilized for two variables, while for more than two variables, an ANOVA with Tukey’s post hoc test was applied. Alternatively, if a non-normal distribution was detected, a Kruskal-Wallis test with Dunn’s post hoc test was employed. All comparative analyses were conducted using GraphPad Prism 8 (GraphPad Software, San Diego, CA, USA). The significance level was established at *p* = 0.05.

## Results

The current investigation assessed and compared the von Mises stresses, strains, and micromovements in the peri-implant region of wide-diameter platform-switched short implants inserted in D4 bone quality, while subjected to immediate loading. A three-dimensional finite element analysis (3D FEA) was employed for this purpose. The implant configurations aimed to restore the absence of a permanent maxillary first molar in D4 bone quality, with a consistent length of 6 mm and varying diameters of 4/3.6 mm, 5/4 mm, and 6/4.8 mm, each possessing specific thread patterns: buttress microthreads, square microthreads, and triangular (V) microthreads. Each model underwent examination using a single applied force of 100 N, exerted at oblique angles (45 degrees) and vertically (90 degrees) simulating immediate loading conditions.

### 4/3.6 mm diameter

**Fig. 6:**
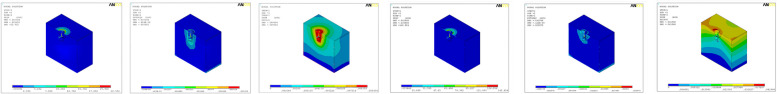
FEA images of 4/3.6 mm diameter buttress threads with 100 N force under vertical and oblique loading for von Mises stress (VMS), strains, and micromovements

**Fig. 7:**
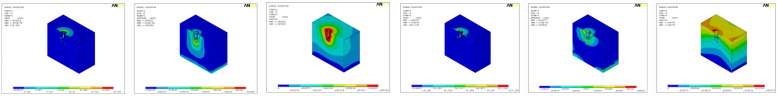
FEA images of 4/3.6 mm diameter square threads with 100 N force under vertical and oblique loading for von Mises stress (VMS), strains, and micromovements

**Fig. 8:**
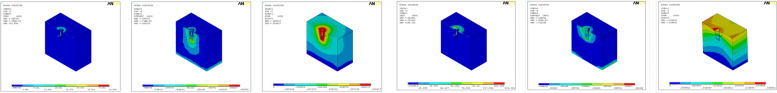
FEA images of 4/3.6 mm diameter triangle threads with 100 N force under vertical and oblique loading for von Mises stress (VMS), strains, and micromovements

**Fig. 9:**
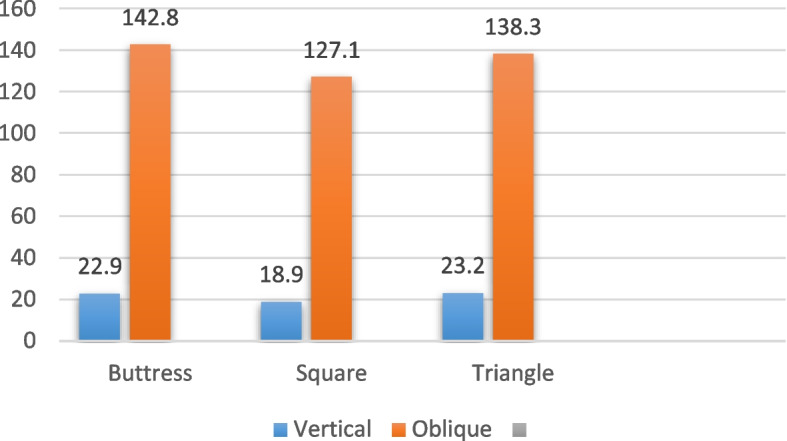
Von Mises stress values around 4/3.6 mm diameter implants in MPa under vertical and oblique loading of 100 N

**Fig. 10:**
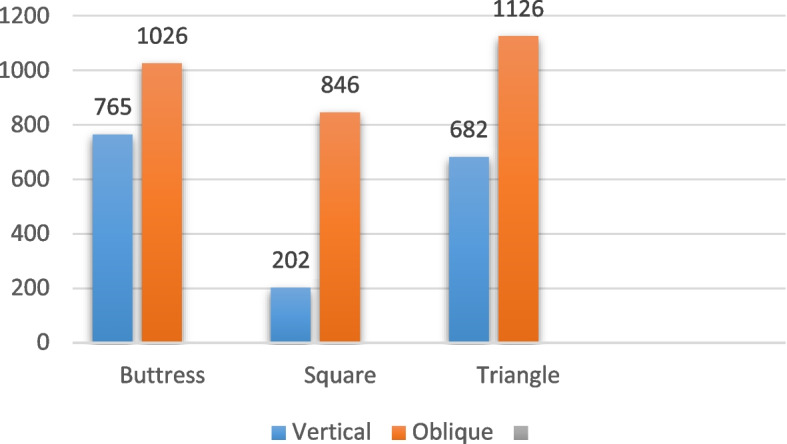
Von Mises strain values around 4/3.6 mm implants in Ɛ under vertical and oblique loading of 100 N

**Fig. 11:**
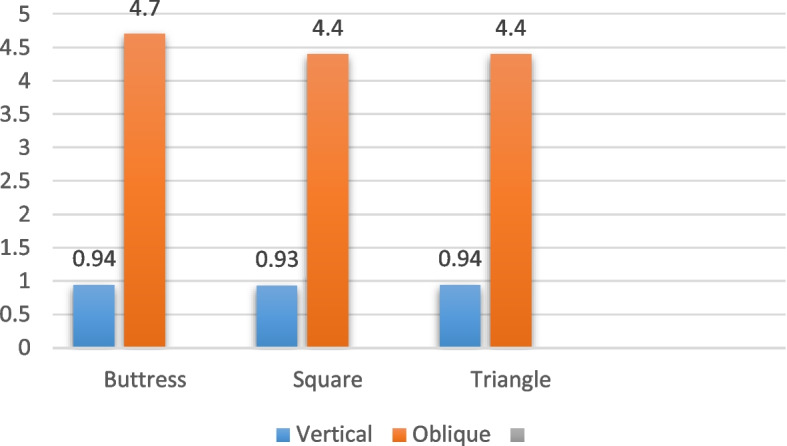
Micromovement values around implants of 4/3.6 mm diameter under vertical and oblique loads of 100 N

Regarding the implants with a diameter of 4/3.6 mm and a length of 6 mm, subjected to vertical loading as presented in Table 4 and illustrated in Figs. 6, 7 and 8, the distribution of von Mises stress at the interface between the bone and the implant was measured. Specifically, for the buttress thread, the stress value was 22.95 MPa, for the square thread, it was 18.9 MPa, and for the triangle thread, it reached 23.2 MPa (as depicted in Fig. 9). In terms of strain, the observations were as follows: the buttress thread exhibited a strain of 765 Ɛ, the square thread displayed a strain of 202 Ɛ, and the triangle thread showed a strain of 682 Ɛ (as demonstrated in Fig. 10). Additionally, the micromovement measurements indicated that the buttress thread experienced 0.94 μm of movement, the square thread demonstrated 0.93 μm, and the triangle thread exhibited 0.94 μm (as indicated in Fig. 11).

**Table 4:** Von Mises stress, strain, and micromovement values at vertical loads of 100 N on 4/3.6 mm diameter implants

	Buttress	Square	Triangle
STRESS (MPa)	22.95	18.9	23.2
STRAIN (Ɛ)	765	202	682
MICROMOVEMENTS (µm)	0.94	0.93	0.94

In the case of implants with a diameter of 4/3.6 mm and a length of 6 mm, when subjected to oblique loading as detailed in Table 5 and depicted in Figs. 6, 7 and 8, the stresses and strains exhibited higher magnitudes. Specifically, the buttress thread showed a stress of 142.8 MPa, the square thread displayed a stress of 127.1 MPa, and the triangle thread exhibited a stress of 138.3 MPa. These values were in contrast to the results under vertical loading, as shown in Fig. 9. The corresponding strain values were 1026 Ɛ for the buttress thread, 846 Ɛ for the square thread, and 1126 Ɛ for the triangle thread, as depicted in Fig. 10. Additionally, micromovements were measured at 4.1 μm for the buttress thread, 4.4 μm for the square thread, and 4.4 μm for the triangle thread, as illustrated in Fig. 11.

**Table 5:** Von Mises stress, strain, and micromovement values at oblique loads of 100 N on 4/3.6 mm diameter implants

	Buttress	Square	Triangle
STRESS (MPa)	142.8	127.1	138.3
STRAIN (Ɛ)	1026	846	1126
MICROMOVEMENTS (µm)	4.7	4.4	4.4

### 5/4 mm diameter

**Fig. 12:**
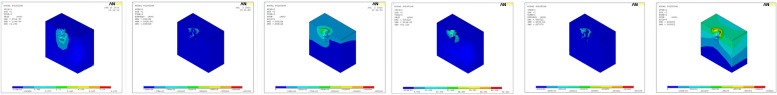
FEA images of 5/4 mm diameter buttress threads with 100 N force under vertical and oblique loading for von Mises stress (VMS), strains, and micromovements

**Fig. 13:**
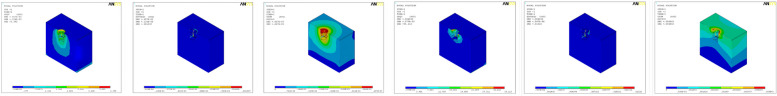
FEA images of 5/4 mm diameter square threads with 100 N force under vertical and oblique loading for von Mises stress (VMS), strains, and micromovements

**Fig. 14:**
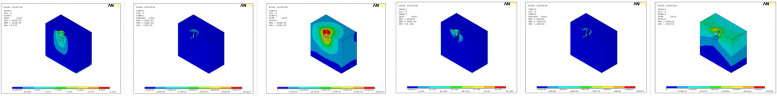
FEA images of 5/4 mm diameter triangle threads with 100 N force under vertical and oblique loading for von Mises stress (VMS), strains, and micromovements

**Fig. 15:**
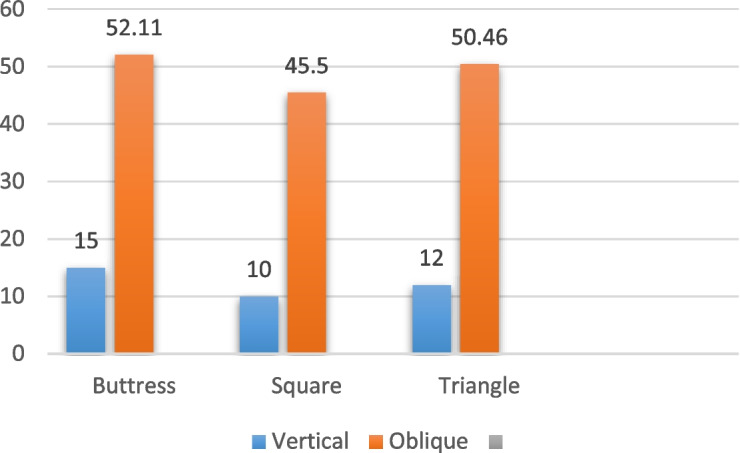
Von Mises stress values around 5/4 mm diameter implants in MPa under vertical and oblique loading of 100 N

**Fig. 16:**
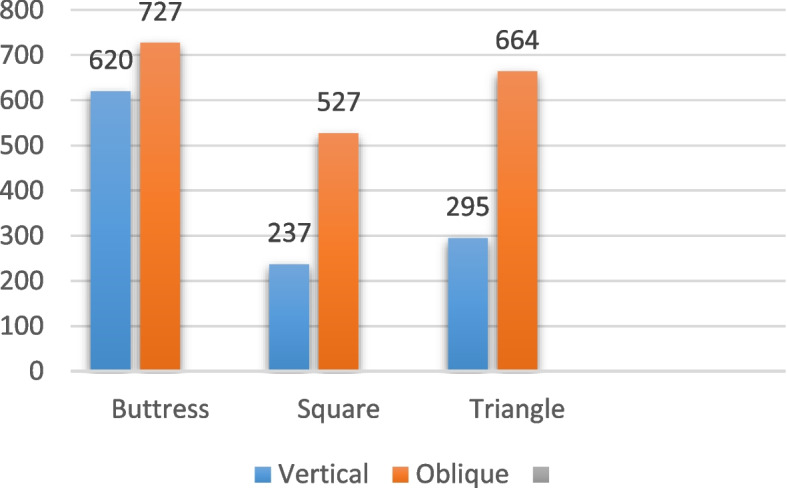
Von Mises strain values around 5/4 mm implants in Ɛ under vertical and oblique loading of 100 N

**Fig. 17:**
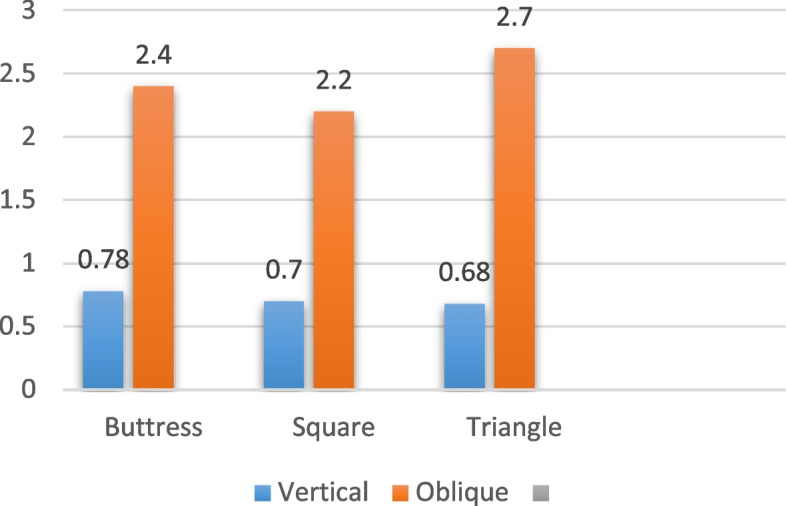
Micromovement values in µm around implants of 5/4 mm diameter under vertical and oblique loads of 100 N at central fossa

For the implants with a diameter of 5/4 mm and a length of 6 mm, subjected to vertical loading (details in Table 6, and depicted in Figs. 12, 13 and 14), the distribution of von Mises stress at the interface between the bone and implant was measured at 15 MPa for the buttress thread, 10 MPa for the square thread, and 12 MPa for the triangle thread (as shown in Fig. 15). The corresponding strains were calculated as 620 Ɛ for the buttress thread, 194 Ɛ for the square thread, and 295 Ɛ for the triangle thread (displayed in Fig. 16). Micromovements were found to be 0.78 μm for the buttress thread, 0.70 μm for the square thread, and 0.68 μm for the triangle thread (illustrated in Fig. 17).

**Table 6:** Von Mises stress, strain, and micromovement values at vertical loads of 100 N on 5/4 mm diameter implants

	Buttress	Square	Triangle
STRESS (MPa)	15	10	12
STRAIN (Ɛ)	620	237	295
MICROMOVEMENTS (µm)	0.78	0.70	0.68

Regarding the 5/4 mm diameter implants measuring 6 mm in length and subjected to oblique loading as detailed in Table 7 and represented by Figs. 12, 13 and 14, the von Mises stresses exhibited distinct values: 52.1 MPa for the buttress thread, 45.5 MPa for the square thread, and 50.46 MPa for the triangle thread (as illustrated in Fig. 15). As for the strains observed, they were quantified at 727 Ɛ for the buttress thread, 527 Ɛ for the square thread, and 664 Ɛ for the triangle thread (depicted in Fig. 16). Additionally, the micromovements recorded were 2.4 μm for the buttress thread, 2.2 μm for the square thread, and 2.7 μm for the triangle thread (depicted in Fig. 17).

**Table 7:** Von Mises stress, strain, and micromovement values at oblique loads of 100 N on 5/4 mm diameter implants

	Buttress	Square	Triangle
STRESS (MPa)	52.1	45.5	50.46
STRAIN (Ɛ)	727	527	664
MICROMOVEMENTS (µm)	2.4	2.2	2.7

### 6/4.8 mm diameter

**Fig. 18:**
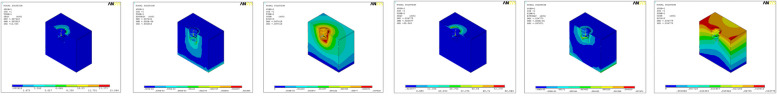
FEA images of 6/4.8 mm diameter buttress threads with 100 N force under vertical and oblique loading for von Mises stress (VMS), strains, and micromovements

**Fig. 19:**
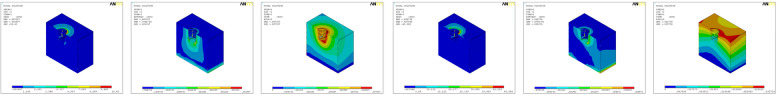
FEA images of 6/4.8 mm diameter square threads with 100 N force under vertical and oblique loading for von Mises stress (VMS), strains, and micromovements

**Fig. 20:**
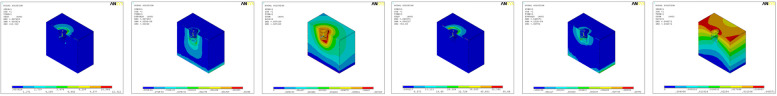
FEA images of 6/4.8 mm diameter triangle threads with 100 N force under vertical and oblique loading for von Mises stress (VMS), strains, and micromovements

**Fig. 21:**
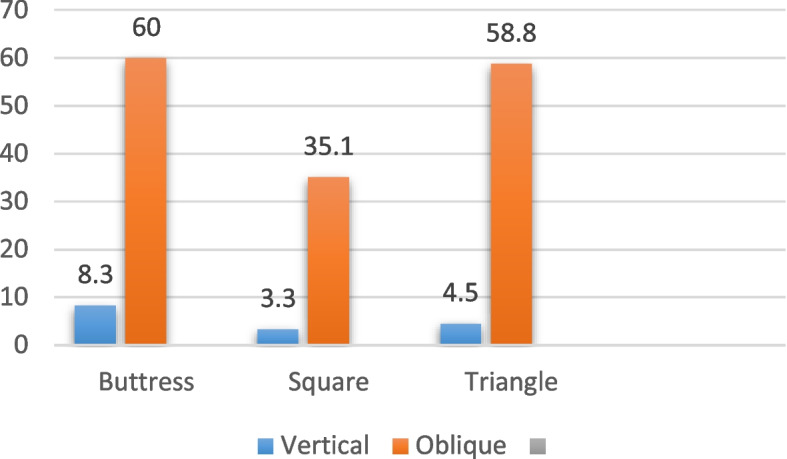
Von Mises stress values around 6/4.8 mm diameter implants in MPa under vertical and oblique loading of 100 N

**Fig. 22:**
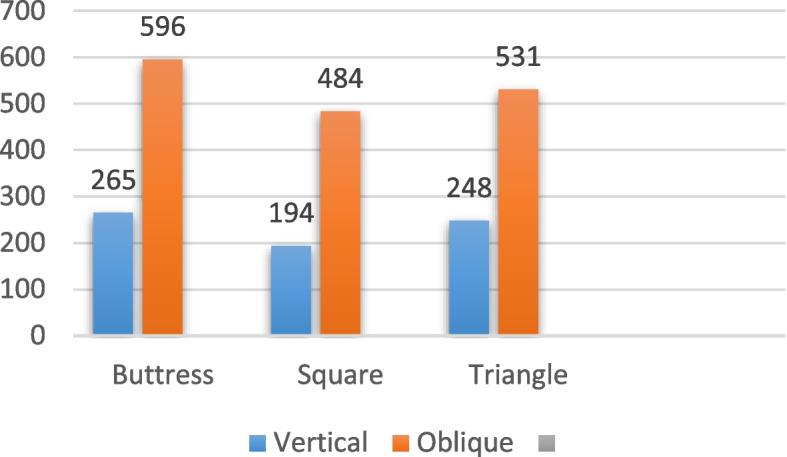
Von Mises strain values around 6/4.8 mm implants in Ɛ under vertical and oblique loading of 100 N at central fossa

**Fig. 23:**
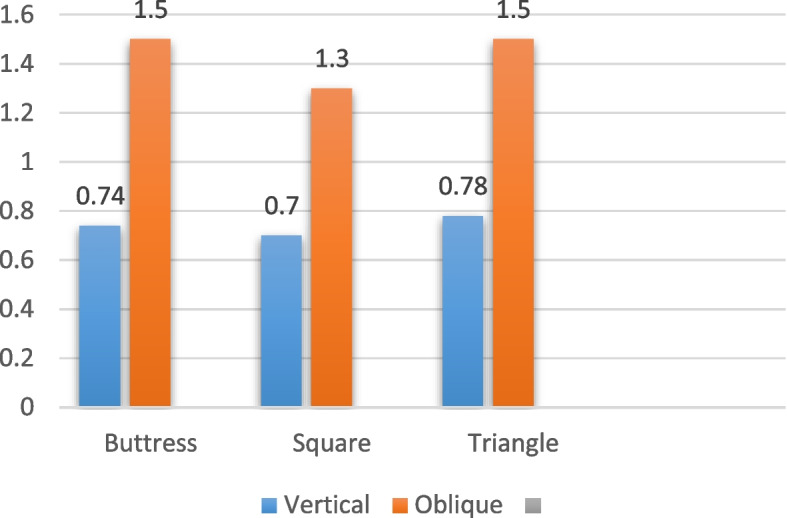
Micromovement values in µm around implants of 6/4.8 mm diameter under vertical and oblique loads of 100 N

In the case of implants with a diameter of 6/4.8 mm and a length of 6 mm, subjected to vertical loading as indicated in Table 8 and illustrated in Figs. 18, 19 and 20, the von Mises stress values around the implant were measured. Specifically, for the buttress thread, the stress was 8.3 MPa, for the square thread it was 3.3 MPa, and for the triangle thread it was 4.5 MPa (as depicted in Fig. 21). The corresponding strain values for these threads were 265 Ɛ for the buttress, 237 Ɛ for the square, and 248 Ɛ for the triangle (as shown in Fig. 22). Moreover, the micromovement values were found to be 0.74 μm for the buttress thread, 0.78 μm for the square thread, and 0.7 μm for the triangle thread (as displayed in Fig. 23).

**Table 8:** Von Mises stress, strain, and micromovement values at vertical loads of 100 N on 6/4.8 mm diameter implants

	Buttress	Square	Triangle
STRESS (MPa)	8.3	3.3	4.5
STRAIN (Ɛ)	265	194	248
MICROMOVEMENTS (µm)	0.74	0.7	0.78

Regarding the 6/4.8 mm diameter implants with a length of 6 mm under oblique loading (refer to Table 9; Figs. 18, 19 and 20), the von Mises stress was measured at 60 MPa for the buttress thread, 35.1 MPa for the square thread, and 58.8 MPa for the triangle thread (see Fig. 21). In terms of strains around the implants, the recorded values were 596 Ɛ for the buttress thread, 484 Ɛ for the square thread, and 531 Ɛ for the triangle thread (shown in Fig. 22). Micromovement values were also obtained, measuring 1.5 μm for the buttress thread, 1.3 μm for the square thread, and 1.5 μm for the triangle thread (depicted in Fig. 23).

**Table 9:** Von Mises stress, strain, and micromovement values at oblique loads of 100 N on 6/4.8 mm diameter implants

	Buttress	Square	Triangle
STRESS (MPa)	60	35.1	58.8
STRAIN (Ɛ)	596	484	531
MICROMOVEMENTS (µm)	1.5	1.3	1.5

### Statistical result

There was an extremely statistically significant result when comparing vertical and oblique stresses (MPa) with 100 N (*p* < 0.001); the greater the stress, the narrower the implant diameter either for vertical or oblique forces. A similar significant result was verified for the strain and micromovements (*p* < 0.001). Moreover, the square thread showed better and more significant stress and strain distribution in the peri-implant bone compared to the other thread designs.

## Discussion

The objective of this study was to evaluate the stress–strain and micromovements of a platform-switched 6 mm short implant with different diameters and threads using a fast-loading protocol. Biomedical mechanical tools such as 3D models, optical elasticity, and strain measurements performed on bone surfaces were utilized, but they have limitations such as complexity and difficulty in modification. FEA introduced by Weinstein et al. has become a popular method in dentistry due to its ease of use and user-friendly interface [38]. This study applied FEA to quantify the von Mises stress, strain, and micromovements of short implants in D4 bone under immediate loading conditions. The FEA study of dental implants must consider both vertical and lateral forces for accurate occlusal direction simulation. A 100 N force was exerted in the direction of the implant axis and at a 45° diagonal angle [39].

Dental implants provide a practical solution for partially or fully edentulous patients, but placement may not be feasible due to reduced bone height or obstacles such as maxillary sinus pneumatization or close proximity to the mandibular canal. A bone augmentation method is employed to enhance the steadfastness of dental implants by amplifying the compactness of native bone [40]. Alaqeely et al. [41] carried out a numerical simulation using the finite element method to assess the stress levels in various areas of the jawbone during conventional drilling (CD) and osseodensification drilling (OD). The OD model exhibited a notable decrease in von Mises stress in comparison to the CD model. This was attributed to areas of increased density in the bone structure, which absorbed a significant portion of the stress and impeded its transmission. Bone augmentation is also advocated but involves multiple procedures and increased postoperative discomfort, cost, and recovery time [22, 42, 43]. An alternative to bone grafting is short implants, defined as less than 7 mm in length [44].

As per Esposito’s findings, the strength and size of the bone are crucial factors in the unsuccessful outcomes of both initial and delayed implant procedures. It is estimated that 50 to 80% of dental implants do not survive in posterior bone regions due to poor bone texture [45]. The Misch classification scheme [30] is widely accepted by clinicians and researchers, dividing jawbone density into four distinct categories based on the classification of trabecular and cortical bone. D1 is dense cortical bone found in the anterior lower jaw, while D2 is thick, dense, and porous cortical bone on the crest with coarse trabecular bone inside, typical of the posterior mandible and anterior maxilla. D3 has thin cortical bone on the crest and fine trabecular bone within, found in the interior/posterior maxilla and posterior mandible. D4, located in the posterior maxilla, is primarily trabecular bone with the thinnest cortical plate. In the FEA simulation, a 0.5 mm cortical plate thickness was applied to D4 bone to replace a missing maxillary molar. Thin cortical bones such as D4 are associated with high stress levels surrounding the implant collar, putting the implant at vulnerability, according to Okumura et al. [46]. Studies by Oliveira et al. [47] and Sevimay et al. [15] showed that the D4 bone generated the highest peri-implant stresses of 170.31 MPa and 180 MPa, respectively.

Immediate loading protocols for dental implants have become more popular due to their advantages such as reduced treatment durations, diminished patient attendance and decreased expenses. compared to the classic two-stage implant placement protocol. These protocols result in improved comfort, function, speech, stability, and psychological factors [48, 49]. This study modeled the interface with the bone of an instantly loaded implant using a friction coefficient of 0.3127 [50] and found favorable stress and strain results around short platform-switched implants.

An implant/crown ratio is unfavorable when short implants are used with longer prosthetic crowns. Short implants can support most prostheses [51, 52]. A remaining vertical bone dimension of 7 mm was presumed for the positioning of the implant in this current investigation. Consequently, considering this presumption, the mean dimension of maxillary 1st molar crowns, which measures 7.5 mm, was presumed to escalate to 13.5 mm within the simulated finite element model. This was carried out to mimic real-world circumstances that account for bone loss. The findings of our investigation aligned with prior studies concerning the utilization of short implants. Bruggenkate and colleagues [53] discovered that short implants exhibited similar rates of survival as long implants during a 1–7 year period. Thread design, pitch, and depth can also improve implant performance and reduce stress, as the compressive stress intensity in the bone structure varies based on the implant thread form. Von Mises and compressive stresses are more prevalent in cortical bone compared to spongy bone. Deporter et al.’s retrospective multicenter report suggested that 5 mm length dental implants may be viable for significantly assimilated posterior areas in cases of partial tooth loss, particularly in the maxilla [54].

In research conducted by Petrie et al. [55], they explored the impact of implant size on stress and strain using FEA. The findings indicated that increasing the implant’s width from 3.5 to 6 mm led to a reduction in crestal strain by a factor of 3.5. Furthermore, extending the implant’s length from 5.75 to 23.5 mm resulted in a decrease in stress and strain parameters by a factor of 1.65. These outcomes imply that the width of the implant significantly controls the stress and strain characteristics. Another study by Himmlova et al. [56] examined implants with lengths ranging from 8 to 18 mm and diameters ranging from 2.9 to 6.5 mm and found that implants with diameters between 3.6 and 4.2 mm had the most significant reduction in stress at 31.5%. While implant length was also considered, it had a less prominent influence than implant diameter. Our study found that using wider diameters of 6 mm in implants can be effective. We observed a decline in both stresses and strains as the diameter underwent an increment from 4 to 6 mm. Upon exposure to vertical loading, the von Mises peri-implant stresses exhibited a substantial reduction: the stress on buttress thread implants decreased by 63.36% from 22.9 MPa to 8.39 MPa, the stress on square thread implants decreased by 82.06% from 18.9 MPa to 3.39 MPa, and the stress on triangle thread implants decreased by 81.07% from 23.2 MPa to 4.39 MPa. When subjected to oblique loading, the stress on 4 mm diameter buttress thread implants was highest at 142.8 MPa.

In contrast, the stress on 6 mm diameter square thread implants was lowest at 35.11 MPa, reducing 75%. Our study found that the maximum strains occurred with 4 mm diameter buttress thread implants under vertical loading (765 Ɛ) and triangular thread implants with a diameter of 4 mm subjected to oblique loading (1126 Ɛ). The implants with 6 mm square thread demonstrated the lowest levels of strains when subjected to both vertical and oblique loading. The strain values recorded were 194 Ɛ and 237 Ɛ for vertical and oblique loading, respectively. These values also demonstrate a reduction of 75% in strain with an increase in the diameter of the implant. In addition, less strain was generated around square thread designs compared to buttress and triangle thread designs. According to these observations, the utilization of wide-diameter implants presents a feasible therapeutic alternative for the sustained upkeep of implant-supported prosthetic rehabilitations in the posterior regions.

Based on the outcomes of our investigation, the von Mises stress, strains, and micromovements displayed consistent elevation when subjected to oblique loading, surpassing those observed under vertical loading across all thread types and diameters. These findings align harmoniously with previous research endeavors. As expounded by Ding et al. [57], oblique loading engenders significantly amplified interfacial stresses and strains compared to vertical loading, owing to the diameter and length’s impact on stress and strain dispersion around the crestal bone encircling the implants. Although a larger diameter and extended implant mitigate stress and strain on the alveolar crest, loading the implant in a buccolingual manner triggers a noticeable augmentation in stress and strain values. Nonetheless, the diameter proves more efficacious than the length in alleviating concentrations of stress and strain in the crestal region. Another FEA investigation [58] revealed that both cancellous and cortical bone endure escalated loads during buccolingual loading. Consequently, heightened stresses in the cancellous bone and implant due to lateral loading may culminate in implant failure. Given this consideration, it is crucial to optimize the design of implant structures to withstand lateral forces optimally.

The implant body is also displaced relative to the surrounding bone due to peri-implant stresses and strains. This type of movement is referred to as micromovement. A significant amount of micromotion may interfere with the osseointegration of the implant [59]. Micromovement of 150 μm should not be exceeded for successful implant healing. There were no micromovements outside these limits in our study’s models. Negligible disparities were registered in the micromotions of the models, with minimal minuscule movements observed for the 6 mm square thread, measuring 0.7 μm, and maximum microshifts for the 4 mm diameter buttress and triangular thread, measuring 0.94 μm, when subjected to vertical loading. The micromotions exhibited greater magnitude under oblique loading for all cases, with the smallest value of 1.3 μm recorded for the 6 mm diameter square thread, and the highest value of 4.7 μm observed for the 4 mm diameter buttress thread under oblique loading. In comparison to 4 mm implants, there was a 26% reduction in micromotions for 6 mm implants, and a 14% reduction for 5 mm implants. Anitua et al. [60] concluded that wider short implants effectively dissipate forces and alleviate stress concentrations, thereby resulting in enhanced stress and strain parameters.

The present study found that square thread implants had the lowest von Mises stress under axial and oblique loads compared to other thread designs. Square and triangle threads also reduced vertical loading stress by 16% and 4%, respectively, compared to buttress threads of 4 mm in diameter. Compared to buttress threads of 5 mm in diameter, square threads reduced stresses by 13% and triangle threads by 4%. Under vertical loading, square threads resulted in a 64% reduction in stress, and triangle threads resulted in a 46% reduction in stress compared to buttress threads of 6 mm in diameter. Similar results were also seen for oblique loading, with square threads experiencing a 42% reduction in stress and triangle threads experiencing a 2% reduction in stress compared to buttress threads of 6 mm in diameter. Alemayehu et al. [19] conducted an extensive FEA study to examine the impacts of five distinct dental implant thread configurations (square, buttress, reverse buttress, trapezoidal, and triangular) on the distribution of stress experienced by the implant and the surrounding bone. The von Mises stresses for the various thread designs around a 4.1 mm x 14 mm implant dimension were recorded as follows: square − 18.75 MPa, buttress − 31.54 MPa, reverse buttress − 33.60 MPa, trapezoidal − 35.53 MPa, and triangular − 51.81 MPa. The researchers concluded that among the thread designs investigated in the study, the square thread model exhibited reduced von Mises stress, shear stress, and displacement magnitude, while simultaneously increasing compressive stress. In a finite element investigation, Mosavar et al. [61] explored the implications of different implant thread configurations on the dispersion of stress within the peri-implant bone. The study encompassed a comprehensive analysis of buttress, reverse buttress, V-shaped, and square thread designs, evaluating their impact on stress distribution patterns in the surrounding bone tissue. A 4 mm × 12 mm implant size was taken into account. Von Mises stresses around the cortical bone were as follows: buttress 47.68 MPa, reverse buttress 47.76 MPa, square 44.50 MPa, and triangular 47.88 MPa. According to the authors’ assertions, the implementation of a square thread configuration exhibited the least amount of stress across all levels of osseointegration within the transitional region between the implant and cortical bone. This region, referred to as the critical zone for design purposes, was utilized in our study. Specifically, for a 4 mm diameter implant, the von Mises stresses were recorded as 18.9 MPa, 22.95 MPa, and 23.2 MPa for square, buttress, and triangular threads, respectively. Our study findings concurred with previous research outcomes. Additionally, due to the implant’s superior stress-carrying capability, followed by the cortical bone, the von Mises stress was comparatively lower in the trabecular bone for all implant models. Chang et al. [62] performed a finite element analysis investigating micromotion patterns involving implants and the surrounding bone, utilizing diverse thread designs such as trapezoidal, buttress, square, and standard V thread. These investigations were conducted under an axial load of 300 N following immediate loading. It was discovered that all micromotion primarily transpired at the interface connecting the cortical and cancellous bone, with the square thread profile demonstrating the most favorable micromotion values. Misch et al. [63] states that square and reverse buttress threads provide excellent primary stability during immediate loading.In contrast to buttress threads, which are efficient at transmitting forces in one direction, square threads are symmetrical and efficient at transferring forces along the screw–thread axis. This superiority of square threads has also been noted in a review by Sennerby et al. [64]. Mosavar et al. [61] used finite element analysis to study four different types of commercial thread-form configurations for solid implants: buttress, reverse buttress, V, and square. They simulated various degrees of osseointegration and assumed that bone was transversely isotropic. The results indicated that square threads produced the best results based on von Mises equivalent stress, pressure, shear stress, and micromotion predictions. However, there are inherent limitations to this study. In this study, finite element analysis was used to simulate the integration of the implant with the surrounding bone and examine peri-implant stresses, strains, and micromovements. The structures in the model were assumed to be homogeneous, anisotropic, and linearly elastic, but it is pertinent to note that living tissues have different properties. This study did not consider the biological aspect, so the mandible and applied loads and muscular action were not considered. When a dynamic force is imposed on the occlusal surface of the prosthesis, it imparts substantial mechanical strain on the implant and the encompassing osseous structure [19]. As a result, the stress patterns observed in this study may not match those seen in clinical applications where dynamic loading is present. Finite element problems are commonly solved using the optimum mesh size. Furthermore, it was not possible to test mesh sensitivity. For a complex three-dimensional problem, a sensitivity study is not possible. It is better to use sensitivity when there is a limited number of elements. Additionally, the present investigation was limited by the absence of numerical model validation using an in vitro experiment to assess the reliability of the results. Since dental studies have a limited load range, sensitive equipment is required. It should be noted, however, that most experimental equipment is available for large-load experiments. A load of less than 100 kg is required for this study, for which it is difficult to conduct experiments. Even though the results of this study were consistent with those obtained in previous studies, they cannot be used to determine a treatment’s reliability [65, 66]. There is a need for further preclinical and clinical research, as well as fatigue testing, for data on aspects such as osseointegration related to the characteristics of fixtures.

## Conclusions

Based on the outcomes uncovered by this investigation, it can be deduced that (i) The implant measuring 6 mm exhibited the least amount of stress, strain, and micro-movements around the implant under both vertical and oblique forces, while the 4 mm implant experienced the highest levels. Increasing the diameter of the implant resulted in a noteworthy reduction in von Mises stress, strain, and micromovements; (ii) Vertical loads generated diminished levels of stress, strain, and micromovements compared to oblique loads; (iii) Subsequent to evaluating all thread configurations under vertical and slanted forces, the square-shaped thread exhibited superior dispersion of stress and strain within the surrounding bone tissue in contrast to the other thread configurations. However, it should be noted that the study did not conduct a mesh sensitivity test or validation test. Therefore, the accuracy and reliability of the results should be interpreted with caution. Further research with additional testing is necessary to confirm these results and ensure their clinical applicability. Overall, this study provides a foundation for future studies on the optimal parameters for short dental implants in cases with limited bone height.

## Data Availability

The datasets used and/or analyzed during the current study are available from the corresponding author on reasonable request.

## Abbreviations

FEA: Finite Element Analysis
VGMs: Virtual Geometric Models
3D FEA: Three-Dimensional Finite Element Analysis

## References

[1] Sahin S, Cehreli MC, Yalcın E (2002). The influence of functional forces on the biomechanics of implant-supported prostheses—a review. J Dent..

[2] Belser U, Buser D, Bernard JP, Lindhe J, Lang NP, Salvi G (2008). Implants in load carrying part of dentition. Textbook of Clinical Periodontology and Implant Dentistry.

[3] Araujo MG, Lindhe J (2018). Peri-implant health. J Periodontol.

[4] Lin C-L, Kuo Y-C, Lin T-S (2005). Effects of dental implant length and bone quality on biomechanical responses in bone around implants: a 3-D non-linear finite element analysis. Biomed Eng Appl Basis Commum.

[5] Brunski JB (1999). In vivo bone response to biomechanical loading at the bone/dental implant interface. Adv Dent Res.

[6] Kawahara H, Kawahara D, Hayakawa M, Tamai Y, Kuremoto T, Matsuda S (2003). Osseointegration under immediate loading: biomechanical stress-strain and bone formation-resorption. Implant Dent.

[7] Brunski JB (1988). Biomechanical considerations in dental implant design. Int J Oral Implantol.

[8] Hansson S (1999). The implant neck: smooth or provided with retention elements. A biomechanical approach. Clin Oral Implants Res.

[9] Stacchi C, Lamazza L, Rapani A, Troiano G, Messina M, Antonelli A, Giudice A, Lombardi T. Marginal bone changes around platform-switched conical connection implants placed 1 or 2 mm subcrestally: A multicenter crossover randomized controlled trial. Clin Implant Dent Relat Res. 2023. epub ahead of print. 10.1111/cid.13186. 10.1111/cid.13186 36725016

[10] Lee DW, Choi YS, Park KH, Kim CS, Moon IS (2007). Effect of microthread on the maintenance of marginal bone level: a 3-year prospective study. Clin Oral Implants Res.

[11] Abrahamsson I, Berglundh T (2006). Tissue characteristics at microthreaded implants: an experimental study in dogs. Clin Implant Dent Relat Res.

[12] Kong L, Liu B, Li D, Song Y, Zhang A, Dang F, Qin X, Yang J (2006). Comparative study of 12 thread shapes of dental implant designs: a three-dimensional finite element analysis. World J Model Simul.

[13] Tada S, Stegaroiu R, Kitamura E, Miyakawa O, Kusakari H (2003). Influence of implant design and bone quality on stress/strain distribution in bone around implants: a 3-dimensional finite element analysis. Int J Oral Maxillofac Implants.

[14] Rungsiyakull P, Rungsiyakull C, Monstaporn M, Sae-lee D, Elsaka S. Effects of bone type and occlusal loading pattern on bone remodeling in implant-supported single crown: a finite element study. J Prosthodont. 2023;1–9. 10.1111/jopr.13679. 10.1111/jopr.13679 36918484

[15] Sevimay M, Turhan F, Kilicarslan MA, Eskitascioglu G (2005). Three dimensional finite element analysis of the effect of different bone quality on stress distribution in an implant supported crown. J Prosthet Dent.

[16] Ichikawa T, Kanitani H, Wigianto R, Kawamoto N, Matsumoto N (1997). Influence of bone quality on the stress distribution. An in vitro experiment. Clin Oral Implants Res.

[17] Al Ahmari NM (2022). Osseo-densification versus conventional surgical technique in low density jaw bone: a split mouth in vivo study. Technol Health Care.

[18] Stacchi C, Troiano G, Montaruli G, Mozzati M, Lamazza L, Antonelli A, Giudice A, Lombardi T (2023). Changes in implant stability using different site preparation techniques: osseodensification drills versus piezoelectric surgery. A multi-center prospective randomized controlled clinical trial. Clin Implant Dent Relat Res.

[19] Alemayehu D-B, Jeng Y-R, Three-Dimensional (2021). Finite element investigation into Effects of Implant Thread Design and Loading rate on stress distribution in Dental Implants and Anisotropic Bone. Materials.

[20] Udomsawat C, Rungsiyakull P, Rungsiyakull C, Khongkhuntian P (2019). Comparative study of stress characteristics in surrounding bone during insertion of dental implants of three different thread designs: a three-dimensional dynamic finite element study. Clin Experimental Dent Res.

[21] Bell RB, Balkey GH, White RP, Hillebrand DG, Molina A (2002). Staged reconstruction of the severely atrophic mandible with autogenous bone graft and endosteal implants. J Oral Maxillofac Surg.

[22] Wallace SS, Froum SJ (2003). Effect of maxillary sinus augmentation on the survival of endosseous dental implants: a systematic review. Ann Periodontol.

[23] Fernandes GVO, Costa BMGN, Trindade HF, Castilho RM, Fernandes JCH (2022). Comparative analysis between extra-short implants (≤ 6 mm) and 6 mm-longer implants: a meta-analysis of randomized controlled trial. Aust Dent J.

[24] Renouard F, Arnoux JP, Sarment DP (1999). Five mm-diameter implants without a smooth surface collar: report on 98 consecutive placements. Int J Oral Maxillofac Implants.

[25] Lazzara RJ, Porter SS (2006). Platform switching: a new concept in implant dentistry for controlling postrestorative crestal bone levels. Int J Periodontics Restorative Dent.

[26] Gardner DM (2005). Platform switching as a means to achieving implant esthetics. N Y State Dent J.

[27] Vela-Nebot X, Rodriguez-Ciurana X, Rodado-Alonso C, Segala-Torres M (2006). Benefits of an implant platform modification technique to reduce crestal bone resorption. Implant Dent.

[28] Canullo L, Rasperini G (2007). Preservation of peri-implant soft and hard tissues using platform switching of implants placed in immediate extraction sockets: a proof-of-concept study with 12- to 36-month follow-up. Int J Oral Maxillofac Implants.

[29] Hurzeler M, Fickl S, Zuhr O, Wachtel HC (2007). Peri-implant bone level around implants with platform-switched abutments: preliminary data from a prospective study. J Oral Maxillofac Surg.

[30] Bidez MW, Misch CE, Misch CE (2005). Clinical Biomechanics in Implant Dentistry. Textbook on Dental Implant Prosthetics.

[31] Jamari J, Ammarullah MI, Santoso G, Sugiharto S, Supriyono T, Permana MS, Winarni TI, van der Heide E (2022). Adopted walking condition for computational simulation approach on bearing of hip joint prosthesis: review over the past 30 years. Heliyon.

[32] Liu F, Mao Z-H, Peng W, Wen S (2022). Biomechanical influence of thread form on stress distribution over short implants (≤ 6 mm) using finite element analysis. Biomed Eng /Biomed Tech.

[33] Rismanchian M, Birang R, Shahmoradi M, Talebi H, Zare RJ (2010). Developing a new dental implant design and comparing its biomechanical features with four designs. Dent Res J.

[34] Lima de Andrade C, Carvalho MA, Bordin D, da Silva WJ, Del Bel Cury AA, Sotto-Maior BS (2017). Biomechanical Behavior of the Dental Implant Macrodesign. Int J Oral Maxillofac Implants.

[35] Ammarullah MI, Santoso G, Sugiharto S, Supriyono T, Wibowo DB, Kurdi O, Tauviqirrahman M, Jamari J (2022). Minimizing risk of failure from ceramic-on-ceramic total hip prosthesis by selecting ceramic materials based on Tresca stress. Sustainability.

[36] Misch CE (1990). Density of bone: Effect on treatment plans, surgical approach, healing, and progressive bone loading. Int J Oral Implantol.

[37] Kang N, Wu Y, Gong P, Yue L (2008). A study of force distribution of loading stresses on implant bone interface on short implant length using 3-dimensional finite element analysis. J Oral Maxillofac Surg.

[38] Geng JP, Tan KB, Liu GR (2001). Application of finite element analysis in implant dentistry: a review of the literature. J Prosthet Dent.

[39] Paracchini L, Barbieri C, Redaelli M, Di Croce D, Vincenzi C, Guarnieri R (2020). Finite element analysis of a New Dental Implant Design optimized for the desirable stress distribution in the surrounding bone region. Prosthesis.

[40] Attanasio F, Antonelli A, Brancaccio Y, Averta F, Figliuzzi MM, Fortunato L, Giudice A (2020). Primary Stability of three different osteotomy techniques in medullary bone: an in Vitro Study. Dent J.

[41] Alaqeely R, AlDosari M, Babay N, Abdulbari A, Ba Hadi A, Ben Yahia F, Abdulghani M. A 3D Finite Element Analysis Study of the Jawbones Response to Osseodensification and Conventional Drilling. In Proceedings of the ASME 2020 International Mechanical Engineering Congress and Exposition, Online, 16–19 November 2020. Volume 5: Biomedical and Biotechnology. 10.1115/IMECE2020-23669.

[42] Raviv E, Turcotte A, Harel-Raviv M (2010). Short dental implants in reduced alveolar bone height. Quintessence Int.

[43] Anitua E, Orive G, Aguirre JJ, Andía I (2008). Five-year clinical evaluation of short dental implants placed in posterior areas: a retrospective study. J Periodontol.

[44] Comuzzi L, Tumedei M, Romasco T, Petrini M, Afrashtehfar KI, Inchingolo F, Piattelli A, Di Pietro N (2023). Insertion torque, removal Torque, and resonance frequency analysis values of Ultrashort, Short, and Standard Dental Implants: an in Vitro Study on polyurethane foam sheets. J Funct Biomater..

[45] Esposito M, Hirsch JM, Lekholm U, Thomsen P (1998). Biological factors contributing to failures of osseointegrated oral implants. Success criteria and epidemiology. Eur J Oral Sci.

[46] Okumura N, Stegaroiu R, Kitamura E, Kurokawa K, Nomura S (2010). Influence of maxillary cortical bone thickness, implant design and implant diameter on stress around implants: a three-dimensional finite element analysis. J Prosthodont Res.

[47] Oliveira H, Brizuela Velasco A, Ríos-Santos J-V, Sánchez Lasheras F, Lemos BF, Gil FJ, Carvalho A, Herrero-Climent M (2020). Effect of different Implant designs on strain and stress distribution under Non-Axial Loading: A three-dimensional finite element analysis. Int J Environ Res Public Health.

[48] Buchs AU, Levine L, Moy P (2001). Preliminary report of immediately loaded huinatural tooth replacement dental implants. Clin Implant Dent Relat Res.

[49] Chow J, Hui E, Liu J, Li D, Wat P, Li W, Yau YK, Law H (2001). The Hong Kong Bridge protocol. Immediate loading of mandibular Branemark fixtures using a fixed provisional prosthesis: preliminary results. Clin Implant Dent Relat Res.

[50] Grant JA, Bishop NE, Gotzen N, Sprecher C, Honl M, Morlock MM (2007). Artificial composite bone as a model of human trabecular bone: the implant-bone interface. J Biomech.

[51] Romeo E, Chisolfi M, Rozza R, Chiapasco M, Lops D (2006). Short (8 mm) dental implants in the rehabilitation of partial and complete edentulism: a 3–14 year longitudinal study. Int J Prosthod.

[52] Maló P, Nobre MA, Rangert B (2007). Short implants placed one-stage in maxillae and mandibles: a retrospective clinical study with 1 to 9 years of follow-up. Clin Oral Implants Res.

[53] Bruggenkate CM, Asikainen P, Foitzik C, Krekeler G, Sutter F (1998). Short (6 mm) nonsubmerged dental implants: results of a multicenter clinical trial of 1 to 7 years. Int J Oral Maxillofac Implants.

[54] Deporter D, Ogiso B, Sohn D, Ruljancich K, Pharoahi M (2008). Ultrashort Sintered PorousSurfaced Dental Implants used to replace posterior teeth. J Periodontol.

[55] Petrie CS, Williams JL (2005). Comparative evaluation of implant designs: influence of diameter, length, and taper on strains in the alveolar crest. A three-dimensional finite element analysis. Clin Oral Implants Res.

[56] Himmlova L, Dostalova T, Kacovsky A, Konvickova S (2004). Influence of implant length and diameter on stress distribution: a finite element analysis. J Prosthet Dent.

[57] Ding X, Liao SH, Zhu XH, Zhang XH, Zhang L (2009). Effect of diameter and length on stress distribution of the alveolar crest around Immediate Loading Implants. Clin Implant Dent Relat Res.

[58] Kitamura E, Stegaroiu R, Nomura S, Miyakawa O (2004). Biomechanical aspects of marginal bone resorption around osseointegrated implants considerations based on a three dimensional finite element analysis. Clin Oral Implants Res.

[59] Laney WR (2008). Glossary of oral and maxillofacial implants, German Edition.

[60] Anitua E, Tapia R, Luzuriaga F, Orive G (2010). Influence of implant length, Diameter, & geometry on the stress distribution: a finite element analysis. Int J Period Rest Dent.

[61] Mosavar A, Ziaei A, Kadkhodaei M (2015). The Effect of Implant Thread design on stress distribution in anisotropic bone with different osseointegration conditions: a finite element analysis. Int J Oral Maxillofac Implants.

[62] Chang PK, Chen YC, Huang CC, Lu WH, Chen YC, Tsai HH (2012). Distribution of micromotion in implants and alveolar bone with different thread profiles in immediate loading: a finite element study. Int J Oral Maxillofac Implants.

[63] Misch CE, Degidi M (2003). Five-year prospective study of immediate/early loading of fixed prostheses in completely edentulous jaws with a bone quality-based implant system. Clin Implant Dent Relat Res.

[64] Sennerby L (2000). Dental implants: matters of course and controversies. Periodontology.

[65] Heboyan A, Lo Giudice R, Kalman L, Zafar MS, Tribst JPM (2022). Stress distribution pattern in zygomatic implants supporting different superstructure materials. Materials.

[66] Kim WH, Lee J-C, Lim D, Heo Y-K, Song E-S, Lim Y-J, Kim B (2019). Optimized Dental Implant Fixture Design for the desirable stress distribution in the surrounding bone region: a biomechanical analysis. Materials.

